# A Methodology for Concomitant Isolation of Intimal and Adventitial Endothelial Cells from the Human Thoracic Aorta

**DOI:** 10.1371/journal.pone.0143144

**Published:** 2015-11-24

**Authors:** Anne Leclercq, Véronique Veillat, Sandrine Loriot, Pirjo Spuul, Francesco Madonna, Xavier Roques, Elisabeth Génot

**Affiliations:** 1 Université de Bordeaux, Bordeaux, France; 2 INSERM, U1045, Bordeaux, France; 3 Assistance Publique-Hôpitaux de Paris, Hôpital Bichat, Paris, France; 4 SFR TransBioMed, Bordeaux, France; 5 Service de chirurgie cardiaque et vasculaire, Hôpital Haut-L’Evêque, Pessac, France; INSERM, FRANCE

## Abstract

**Background:**

Aortic diseases are diverse and involve a multiplicity of biological systems in the vascular wall. Aortic dissection, which is usually preceded by aortic aneurysm, is a leading cause of morbidity and mortality in modern societies. Although the endothelium is now known to play an important role in vascular diseases, its contribution to aneurysmal aortic lesions remains largely unknown. The aim of this study was to define a reliable methodology for the isolation of aortic intimal and adventitial endothelial cells in order to throw light on issues relevant to endothelial cell biology in aneurysmal diseases.

**Methodology/Principal Findings:**

We set up protocols to isolate endothelial cells from both the intima and the adventitia of human aneurysmal aortic vessel segments. Throughout the procedure, analysis of cell morphology and endothelial markers allowed us to select an endothelial fraction which after two rounds of expansion yielded a population of >90% pure endothelial cells. These cells have the features and functionalities of freshly isolated cells and can be used for biochemical studies. The technique was successfully used for aortic vessel segments of 20 patients and 3 healthy donors.

**Conclusions/Significance:**

This simple and highly reproducible method allows the simultaneous preparation of reasonably pure primary cultures of intimal and adventitial human endothelial cells, thus providing a reliable source for investigating their biology and involvement in both thoracic aneurysms and other aortic diseases.

## Introduction

Human thoracic aneurysm of the ascending aorta (TAA) is a chronic, asymptomatic and potentially lethal disease. It is characterized by dilatation of the aortic wall, which can progress to vessel dissection and rupture. Prophylactic surgical repair remains the most effective treatment modality but does not prevent disease progression. There is therefore an urgent need to understand the mechanisms underlying the pathogenesis of the disease in order to develop alternative therapies for treating the causes of human TAAs.

Clinically, arterial enlargement is attributed to unbearable wall tension superimposed on defective aortic wall integrity and impaired aortic repair mechanisms. The wall of the aorta consists of three well-defined layers [1]: *i)* the intima, which is the innermost layer composed of the endothelium whose cells rest and adhere onto a basement membrane; *ii)* the media which consists of ~70 layers of alternating elastic laminae [2] and embedded smooth muscle cells (SMCs), collagen fibers, proteoglycans (PG) and glycosaminoglycans (GAG); and *iii)* the tunica adventitia, the outermost and most complex layer, which is composed of a variety of cells and connective tissue where collagen fibers stabilize and anchor the aorta to adjacent tissues [1]. Aortic tissues receive nourishment and waste removal by diffusion of fluids from the vessel lumen. Additionally, when vessel wall thickness exceeds a certain critical magnitude, as for the thoracic segment, small arteries and veins, the *vasa vasorum* (VVs), supply the cells of the outer parts of the aorta [3–5].

Whatever their etiologies, most TAAs present the common features of elastic and collagen fiber fragmentation or degradation [6,7], SMC rarefaction and accumulation of zones of mucoid degeneration [8–10]. To date, the leading role has been attributed to SMCs becoming subject to massive apoptosis. Recent studies have demonstrated altered regulation of TGFbeta signaling in both syndromic and non-syndromic TAAs which, by affecting SMC survival and extracellular matrix (ECM) integrity, are thought to play a major role in the progression of the disease [8]. Without questioning these established findings, new data showing major effects of TGFbeta on endothelial cells (ECs) suggest that these cells are plausible players in the process [11]. Supporting this hypothesis, ECs were shown to play the triggering role in the formation of aneurysms in the ascending portion of the thoracic aorta in an experimental model of angiotensin II (Ang II)–infused mice where the AngII type 1 (AT1) receptor carried by these cells initiates the pathological cascade [12]. Other studies now show a pivotal role for EC-derived reactive oxygen species (ROS) in determining the susceptibility of the aortic wall to AngII-mediated aortic dissections [13]. In addition, recent studies point to the VVs as being important players in triggering the process [14]. Microvessel density is increased in the adventitia of TAAs as compared to healthy aortas [15]. There is thus a need for a reliable protocol to isolate adventitial ECs in parallel with the intimal ones, to perform basic research in order to better understand their role in aneurysmal diseases [16].

Various procedures are commonly applied for EC isolation: *i)* enzymatic digestion with collagenase which is useful to detach the ECs from the basement membrane and is mainly used for large vessels [17]. Amongst the advantages of the technique are a good yield and a high purity but detrimental effects on certain plasma membrane proteins may be induced with this approach; *ii)* mincing and enzymatic digestion, followed by cell selection, using an appropriate culture medium or cell sorting using specific antibodies or a combination of antibodies (the choice of the antibodies largely depends on the target tissue), a costly and low-yield technique often used for small vessels [18] which does not take into account possible alterations in endothelial marker expression associated with the pathological state and/or vascular bed origin; *iii)* mechanical scraping involving physical detachment for macrovascular ECs. When handled properly, the latter approach causes little cell damage and provides an EC population of reasonable purity but with low yields. These isolation procedures involve culturing and subculturing of cells which in combination with the complete loss of the native environment may affect EC phenotype [19]. Such approaches thus require validation by additional immunohistochemical analyzes.

Considering the heterogeneity of EC populations (micro- and macrovascular cells) within the vessel [20–22] and the fact that the pathological state may alter cell surface marker expression [20,21], we sought to develop a technique that could limit the risk of selecting EC subpopulations. Therefore, we ruled out antibody-based sorting approaches [23] and avoided all approaches involving positive selection. We developed a method to isolate intimal ECs (IECs) and adventitial ECs (AECs) simultaneously from the same human aortic segment. By using cell culture techniques, together with a series of complementary tests including cell morphologic analysis and immunophenotyping [24–27], we have developed a reliable procedure with which ECs can be successfully isolated, amplified and become amenable to biochemical and functional assays in order to study their involvement in aortic diseases.

## Materials and Methods

### Tissue samples

Aneurysmal aortic samples (n = 20) were obtained from patients undergoing vascular replacement surgery with the Bentall procedure at the Cardiovascular Surgery Unit (University Heart Hospital Bordeaux, France). Non-aneurysmal aortic tissue samples (n = 3) were obtained from patients who had died in accidents and who were used as heart graft donors. All tissues samples, originating from either patients or healthy donors (n = 23) are considered as surgical waste in accordance with French ethical laws (L.1211 to 3 to L.1211 to 9). All tissues samples, originating from either patients or healthy donors (n = 23), were obtained from the surgeons F. M. and X. R and anonymised before collection. The study was approved by the local governmental Human Research Ethics committee (Ministère de l’Enseignement Supérieur et de la Recherche (MESR)-2013-1941)). Representative examples are shown to illustrate the procedure but all approaches were applied to the 23 patients/donors of the study.

### Human Aortic Endothelial Cell isolation

Tissues were collected in saline and quickly processed after surgery. Aortic microvascular ECs were isolated from the adventitia (AECs) and aortic macrovascular ECs from the intima (IECs). Aortas were cut longitudinally along the convex and concave curvature yielding two symmetrical open segments. Both pieces were processed in parallel for isolating the two types of ECs independently from the same surgical sample. Prior to enzymatic digestion, a small piece of tissue was cut out and was either paraformaldehyde (PFA)-fixed then paraffin-embedded, or OCT-embedded, for histological analysis. Next, both main segments were immersed in a limited volume (5 ml in a 10 cm diameter dish) of PBS containing 0.1% collagenase (Collagenase D, C5138, Sigma) so that only the (targeted) side in contact with the bottom of the dish (the intimal one in the first sample and the adventitial one in the second), was exposed to the collagenase action. The samples were then incubated for the time indicated at 37°C in a moist incubator in 5% CO_2_. ECs released under these conditions were collected by rinsing the collagenase-exposed side of the aortic segments with serum-free EGM-MV (PromoCell). The detached material was centrifuged and seeded in complete medium (EGM-MV with serum) in one well of a 4-well plate containing a glass coverslip. This procedure was repeated several times (4 to 16 rounds), each well corresponding to one round of collagenase action. Contaminating red blood cells were discarded daily by gentle PBS washing. On the fourth day after cell isolation, cells had spread and were then left to grow on the coverslip and around. They were then examined by phase contrast. Once reaching a subconfluent state, the coverslips were removed and immersed in a 4% (w/v) PFA solution (Electron Microscopy Sciences) in PBS to fix the cells. For each well, the remaining cells were left to repopulate the surface freed by removing the coverslip until confluence was reached. Control commercial bovine aortic endothelial cells (BAEc) were purchased from Lonza and cultured in the same medium.

### Human Aortic Endothelial Cell culture

Immediately after the collagenase step, the freshly isolated cells were seeded together with the matrix debris generated by the action of the collagenase on non-coated plastic dishes and maintained in EGM-MV (containing endothelial cell growth complement, EGF 10 ng/ml, heparin 90 μg/ml and hydrocortisone 1 μg/ml) supplemented with 5% fetal calf serum and 1% antibiotics (10000 U Penicillin/ml and 10000 U Streptomycin/ml, Lonza). This medium was developed by PromoCell for culturing human ECs from either microvascular or macrovascular origin. HUVEC cells were purchased from Lonza (# cc-2817) and cultured in EGM2-MV medium (Lonza). Cells were passaged at confluence after treatment with trypsin-EDTA buffer (170000U trypsin/l, Lonza). Confluence is mimicking the native *in vivo* state of ECs. All cells were used at passages 0–2. TGFbeta concentration in the medium, provided by the serum, was measured with the ELISA assay (Ready-Set-Go, human/mouse TGFbeta, 2^nd^ generation, eBioscience). Phase contrast images of the isolated cells in culture were acquired with a Nikon Eclipse TE 2000-E inverted microscope with a 10x air objective.

### Histology

Pieces of intact aortic tissues were fixed in 4% (w/v) PFA in PBS for 48 hours and then processed for routine paraffin-embedding or were directly embedded in OCT. Aortic wall specimens were oriented to obtain cross-sections perpendicular to the axis of blood flow. Five/ten micrometer-thick serial sections were routinely stained with either hematoxylin/eosin (paraffin) or Sirius Red (OCT), respectively. Sections stained by Sirius Red were analyzed by fluorescence microscopy (Nikon Eclipse TE2000-E) in order to observe collagen and elastic fibers simultaneously [28]. The fluorescence signal of this histochemical dye staining collagens was detected with the DS red excitation fluorescence filter which has a 50 nm excitation band covering the 555/585 nm spectral range. Elastin autofluorescence was detected with a FITC filter with a 30 nm excitation band covering the 465/495 nm spectral range. Serial sections of these tissues were also used for immunohistochemical staining.

### Immunohistochemistry

Immunohistochemistry was performed on OCT cryosections or paraffin-embedded sections of 23 representative aortic specimens using a polyclonal rabbit anti-human vWf (Dako, #A0082) as primary antibody diluted to 10 μg/ml in TBS/TC (Tris-Buffered Saline—0.2% Tween20–0.6% casein, pH6.0) and a peroxidase LSAB-DAKO kit (Dako) for detection. The binding reaction was detected by DAB (3,3'-diaminobenzidine). Slides were then counterstained with Nuclear red (nucleus)/Alcian blue (proteoglycans). Irrelevant control antibodies (Dako) were applied at the same concentration in order to assess non-specific staining.

### Immunocytochemistry

For immunofluorescence labeling cells were fixed with PFA 4% (w/v) in PBS for 30 minutes. PFA was quenched using 50 mM glycine (pH 8.0). Cells were subsequently washed in PBS and permeabilized with 0.1% triton X-100 (w/v) in PBS for 5 minutes. The samples were blocked with Image-iT^TM^ (Invitrogen) for 30 minutes. The cells were then washed with PBS and primary antibodies were applied for 1 hour: a polyclonal rabbit anti-human vWf (Dako, #A0082) at 7.75 μg/ml, a polyclonal sheep anti human vWf (Serotec, #AHP062) at 2 μg/ml, a polyclonal rabbit anti VE-cadherin (Cell Signalling, #D87F2) at a 1/400 dilution and a monoclonal mouse anti-CD31 (BD Pharmingen, 30881A) at 10 μg/ml. After washing with PBS, secondary antibodies were applied also for 1h, namely Alexa-Fluor®-conjugated donkey anti-mouse, rabbit or sheep IgG (all from Jackson ImmunoResearch Laboratories Inc). Slides were mounted using ProLong gold antifade reagent with DAPI (Invitrogen). Series of optical sections were acquired using a confocal LSM 510 (Carl Zeiss Microimaging) with a 63x (numerical aperture [NA], 1.4) oil immersion objective.

### Immunoblot analysis

Aortic EC protein extracts were obtained from cell confluent monolayers that had been exposed or not to recombinant human TGFbeta (5ng/ml; R and D systems) using Laemmli buffer. Proteins were separated by SDS-PAGE and transferred after electrophoresis onto a PVDF membrane (Millipore). Samples were blocked in 5% (w/v) BSA in TBS/Tween 0.1% (w/v). The membrane was probed with either a polyclonal antibody against eNOS at 1/1000 (Cell Signaling Technology, #9572), a polyclonal anti-VE-cadherin antibody at 1/100 (Cell Signalling Technology, #2500), a polyclonal anti-CD31 antibody at 0.4 μg/ml (Santa Cruz, sc-1506), a monoclonal anti-smooth muscle myosin heavy chain (SM-MHC) antibody at 1μg/ml (Chemicon, MAB3570), a monoclonal anti-alpha smooth muscle actin (alphaSMA) antibody at 1/2000 or 2.5 μg/ml (Sigma, A5228 or A2547) or a monoclonal anti-tubulin antibody at 0.2 μg/ml (Sigma, T6074). In a second step, the species corresponding to horseradish peroxidase-conjugated secondary antibodies (from Jackson ImmunoResearch Laboratories Inc) were applied. The immunoreactions were developed using the Amersham ECL^TM^ (Enhanced Chemi-luminescence) Western blotting detection reagents (GE Healthcare) and the membranes treated with the detection reagent were exposed to Amersham films (GE Healthcare).

### *In vitro* tube formation assay on Matrigel

*In vitro* tube formation assays were performed in a pre-chilled 48-well plate with 100 μl of growth factor-reduced Matrigel (BD Biosciences) that was allowed to gelify at 37°C for 30 min. Thereafter, 2×10^4^ intimal or adventitial aortic ECs from 11 different patients were seeded in triplicate onto the Matrigel in EGM-MV media containing 5% (v/v) FCS, incubated at 37°C for 16–18 h to allow formation of tubular structures and further analyzed by microscopy using a Nikon Eclipse TE2000-E (Nikon Ltd) at 4x magnification. The angiogenic response was measured by image analysis. The closed polygons and branching points formed in 5 random microscopic fields per well were counted manually and values averaged.

### Statistical analysis

Differences between IECs and AECs from the same aortic sample were evaluated by the Wilcoxon paired non-parametric test. Differences between aortic samples were assessed by the Mann-Whitney non-parametric test [29]. Statistical significance was accepted for p<0.05. When appropriate, results were expressed as box plots, in which the median is shown. Upper and lower limits of boxes represent interquartiles (25^th^ and 75^th^), whereas upper and lower bars show percentiles (10^th^ and 90^th^).

## Results

### Overall features of an aneurysmal aorta

Prior to focusing on the endothelium, we first examined features of the diseased aorta by analyzing its main matrix components. We used microscopy to image cryostat sections from aorta stained with Sirius Red (see Material and Method section). Combined with elastin autofluorescence, this approach allowed us to visualize the structural organization of collagens and elastin in the tunica media (S1A Fig). Within the structured wall consisting of layers of alternating elastic laminae and collagen fibers, several foci showed severe disorganization of elastic lamellae with areas of complete disappearance of the elastic fiber network (S1A Fig).

The next objective was to highlight the location of ECs across the aortic section. The expression pattern of two common EC markers (vWf and CD31) was evaluated in the context of TAAs, because ECs from different types of vessels express EC antigens heterogeneously. The CD31 staining appeared discontinuous on the intimal side (data not shown). This was not the case for vWf staining which displayed a continuous pattern in all cases. Regarding the VVs, vWf staining detected more vessels than CD31 did in the outer third of the media and in the adventitia (data not shown). Therefore, the aneurysmal aorta was immuno-stained with vWf and counterstained with Alcian blue and Nuclear red. Alcian blue, a polyvalent basic dye forming complexes with PG and GAG, was used to detect mucoid basophilic material corresponding to the zone of cystic medial degeneration (S1B Fig). For patient 1, VVs were found in the vicinity of mucoid degeneration zones highlighted by intense Alcian blue staining [7].

We next focused on ECs that we aimed at isolating from this material. We first considered the structure of the aorta in order to devise the best technique for isolating and culturing the cells of interest simultaneously, namely IECs and AECs. The study published by Stary in 1992 [1] gives a reference description of the structural organization and functionality of the aortic intima (S1C Fig). Even in normal healthy subjects, the thickness of the intima is not uniform [1]. Regions of higher thickness correspond to physiological adaptation to changes in flow and wall tension. Nevertheless, IECs represent the principal cell type of the intimal compartment. This tunica also includes a PG layer containing an abundance of finely reticulated non-fibrous connective tissue. Elastic fibers are scarce and SMCs are sporadically found. A thicker layer underlying this PG mixture contains elastic fibers, collagens and synthetic SMCs. In the outer part of the aorta, ECs line the VVs which are found in the adventitia and outer and middle thirds of the media [2]: the inner third is thus avascular. The VVs consist of a network of small arteries flanked by two small veins providing an entire microvascular bed within the wall of the aorta. Capillary vessels have also been noted. VVs are surrounded by SMCs, fibroblasts, immune modulatory cells, progenitor cells as well as peripheral adrenergic nerves (S1D Fig). Major components of the adventitial ECM produced by fibroblasts are types I and III fibrillar collagens [30]. IECs, which are the innermost layer lining the central lumen of the aorta, lend themselves to a straightforward cell isolation approach but contamination by other cell types may occur [31]. The isolation strategy for the VV ECs of the adventitia (without elastase the EEL remains intact, so the media is not reached) and external part of the media (AECs) appears more challenging due to the architectural complexity and cell type diversity of the adventitia.

### Concomitant isolation of ECs from the intima and adventitia of aortic vessel segments

**Fig 1:**
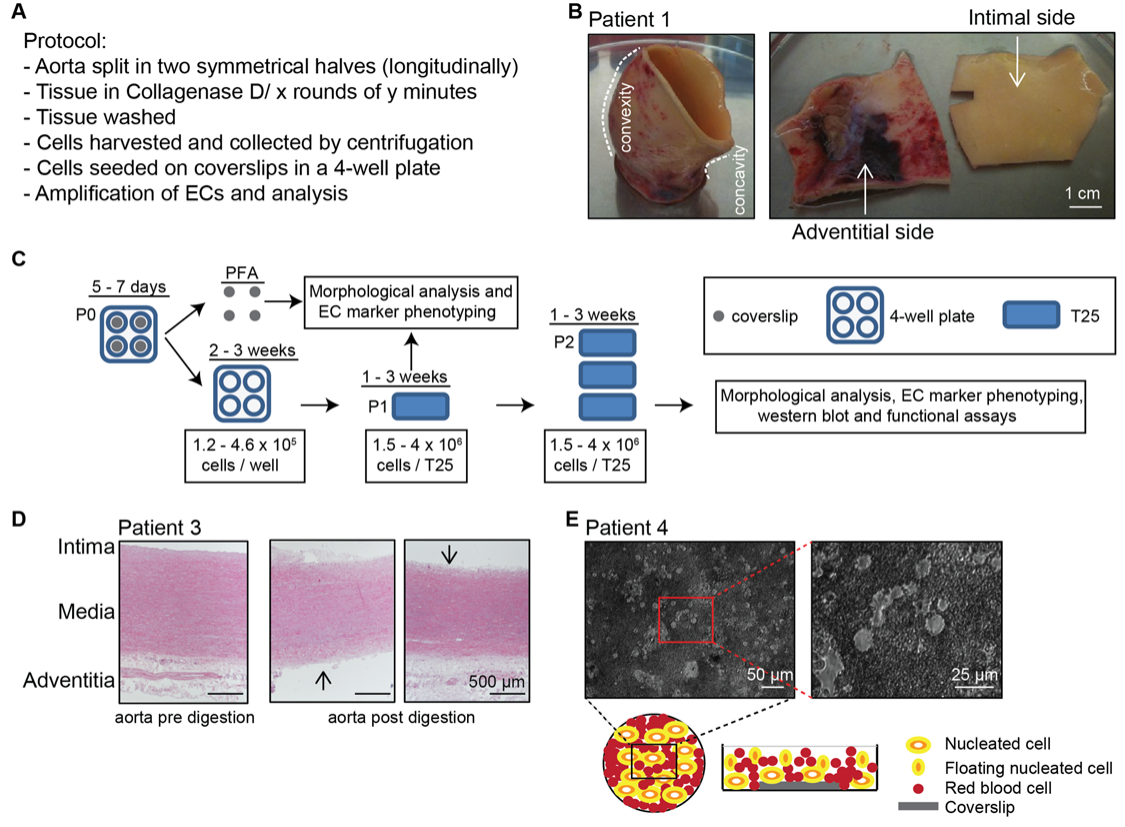
Overview of the sequential enzymatic digestion procedure for isolating cells from human aortic tissues. (**A**) The various steps of the isolation protocol are displayed. (**B**) A representative aneurysmal aorta segment is shown before and after longitudinal sectioning from its convex to its concave border. (**C**) A schematic diagram summarizes the fractionation procedure from the first round of enzymatic digestion (P0) to the expanded population (P2). (**D**) Histological analysis (H and E) of the representative aorta of patient 3, before and after collagenase digestion of the intimal or adventitial sides shows the efficiency of the enzyme action (4 rounds, 10 minutes each). (**E**) Phase contrast image of the crude cell population isolated after the first round of enzymatic digestion seeded in the 4-well plate (T0). Below left, a schematic representation of these cells seen from the top and from the side with the unattached cells floating in the culture medium.

The aorta was cut along its longitudinal axis yielding two symmetrical open segments (one luminal side up and the other adventitial side up) processed in parallel for isolating the two types of ECs independently from the same sample (Fig 1A and 1B). Considering the matrix components surrounding aortic IECs and AECs (S1 Fig), type D collagenase was chosen for EC isolation. This bacterial enzyme with substrate specificity broader than the vertebrate ones, is able to proteolyse almost all collagen types, creating multiple cleavages within the triple helical region. Collagenase D was applied to detach the cells gently. The material washed away from the proteolysed side of the aorta was collected and centrifugated. The pellet, containing the detached cells and the insoluble fragments of the native ECM as a result of the collagenase action, was resuspended in complete medium to seed a dish where one coverslip had been set to assess the specificities of cells obtained immediately after the isolation step (hereafter referred to as P0). Once fixed (between d5 and d7), cell morphology (examined by phase contrast microscopy), EC marker expression (examined after immunolabeling by immunofluorescence microscopy) and other criteria of interest that may be altered upon culturing could be analyzed and compared with those of cells obtained after passaging to be used for biochemical characterization and functional assays (Fig 1C). Collagenase application was repeated several times (4 to 16 rounds) in order to obtain at least one EC-enriched fraction originating from each intimal or adventitial tunica.

After the last round of collagenase D treatment, for some patients, the remaining tissue sample was fixed in PFA and paraffin- or OCT-embedded (or both when two segments were available) for further histological comparison with the initial segment (processed before treatment) and visualization of the action of collagenase. Fig 1D shows the histological examination of patient 3 aorta, before and after enzymatic digestion on the collagenase-exposed side of each aorta segment. The adventitia clearly appeared digested up to the external elastic lamina (EEL) adjacent to the media. This indicated that, for this patient, the vasculogenic zone (displayed in S1D Fig) rich in VVs was reached during the rounds of enzymatic digestion. The intimal side appeared intact. On the other hand, when the intima was subjected to enzymatic digestion, the media appeared untouched, the collagenase being unable to lyse the elastic fibers and the adventitia remaining intact.

On the day after, cells of the highest density had selectively attached (Fig 1E), i.e., transparent round cells (hereafter referred to as P0 cells). Small round cells with a brown appearance, i.e., red blood cells were sedimented at the bottom of the well. The supernatant containing floating cells and non-attached cells was harvested every day and discarded until the transparent round cells had spread. Once P0 cells reached near confluence, the coverslip was harvested and fixed. In the initial dish, cells were left to expand then were transferred to a T25 flask (hereafter referred to as P1 cells) (Fig 1C). Once P1 cells reached near confluence, the population was detached and split into 3 sets (hereafter referred to as P2 cells) (Fig 1C) for morphological analysis and EC marker immuno-phenotyping, western blot analysis and functional assays.

### Characterization of isolated cells

**Fig 2:**
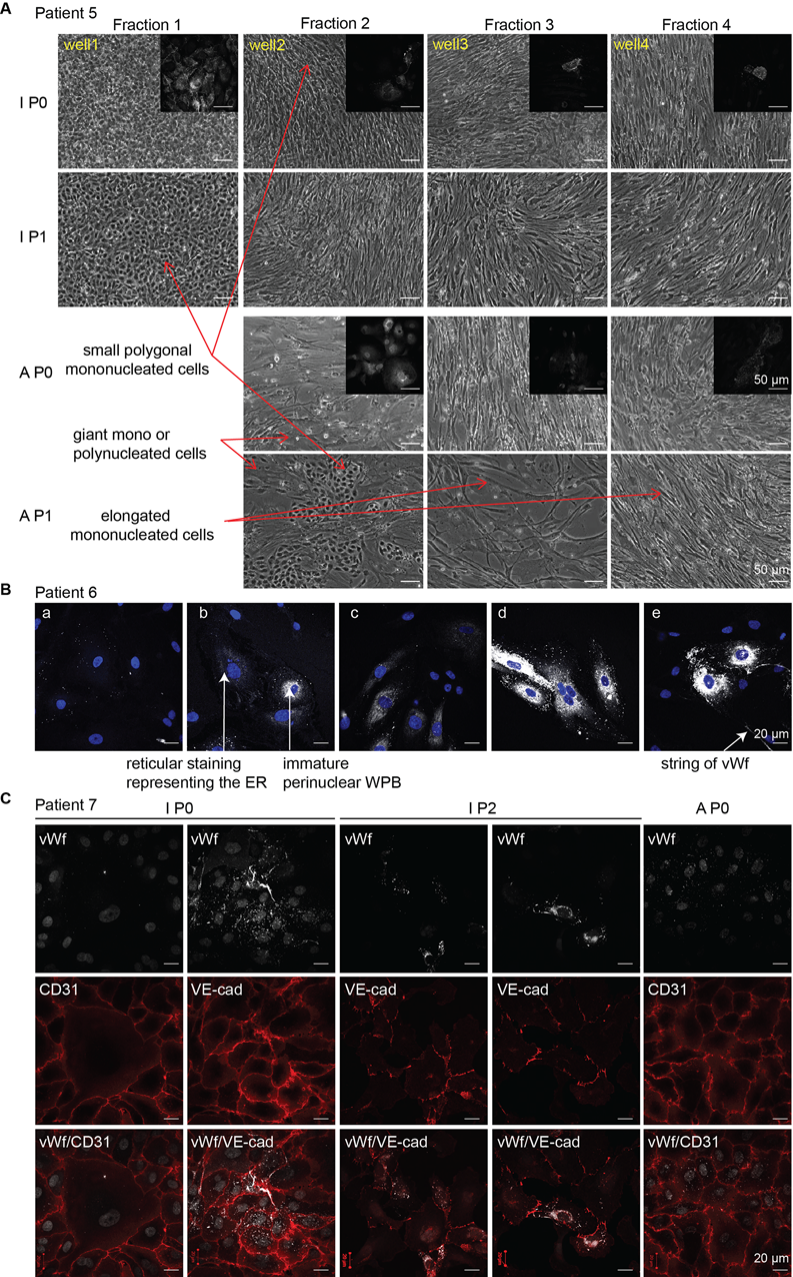
Characterization of the isolated cells. **(A)** Representative phase contrast micrographs show the cell population collected at each of the four consecutive rounds of digestion (well 1 to 4) on the intimal (I) and adventitial (A) side of the aorta. In both cases, three main phenotypes were observed: small polygonal cells forming areas of cobblestone appearance, giant mono- or multinucleated cells and elongated cells. All three phenotypes were also detected at P1. Immunofluorescence analysis performed on each P0 population identified ECs by positive vWf staining (insert). The small polygonal mononucleated cells and the giant mono- or polynucleated morphologies, stained both for vWf. Rare elongated cells could also be vWf positive, their unusual shape likely being due to surrounding peer pressure. Magnification of the phase contrast picture**s** and immunofluorescence images is 10x and 63x, respectively. (**B**) Immunofluorescent micrographs showing the diversity of vWf presentation. vWf staining performed on adventitial aortic endothelial cells shows diverse and typical vWF staining patterns such as that of elongated WPBs throughout the cell body (d and e), residual vWf granules after degranulation of WPB or seen in cross section (a), reticular vWf around the nucleus corresponding to vWf re-synthesis after degranulation (b and c) or extracellular string of vWf (e). (**C**) Immunofluorescent micrographs of isolated cells double-stained for vWf together with either CD31 or VE-cadherin validates vWf staining. Small polygonal mono-nucleated cells and most of the giant mono- or polynucleated vWf positive cells also stained for CD31 or VE-cadherin at P0 and P2 and in IEC and AEC.

Next, we further characterized the P0 cells isolated from the intimal and adventitial sides. These cells were the ones of the highest density selected by plating a dense cell population on a limited surface. By phase contrast microscopy, three types of morphology were observed: the main population consisted in small polygonal cells forming areas of cobblestone appearance (Fig 2A), a characteristic feature of cultured ECs, and was therefore referred to as “small polygonal cells” (thereafter IECs). The second cell population consisted in large, mono- or multinucleated cells and was referred to as “giant cells” (Fig 2A). The third cell population consisted in elongated cells exhibiting a more fibroblast-like morphology cells and was referred to as “elongated cells” (Fig 2A). Depending on the specimen and number/duration of enzymatic digestion rounds (see below), the proportion of the three phenotypes varied from a virtually pure population of the small polygonal mononuclear cells to a combination of the two or all phenotypes in various proportions. Similar morphological results were observed from both adventitial and intimal isolated cells. Cells retained these phenotypes after the first passage. The majority of ECs in culture displayed the typical “cobblestone” appearance at confluence, distinguishing them from contaminating cells isolated from the aortic tissue during the process. When issued from adventitial samples, wells could occasionally be found devoid of cells (Fig 2A), due to the heterogeneity and scarcity of VVs in some areas of the adventitia (see S1D Fig).

The next step was to characterize the cells of endothelial origin amongst the isolated cell population by immuno-phenotyping (Table 1) [22]. The coverslip was taken off the well and processed for vWf immunofluorescent staining while the remaining cells were left to populate the space freed for amplification. Fig 2A (insert) shows that both the small polygonal mononucleated cells and the giant mono- or polynucleated cells were positive for this EC-specific marker, irrespective of their intimal or adventitial origin (Table 1) [22]. Rare elongated cells were occasionally found positive. Interestingly, the adventitial ECs seemed to displayed fewer Weibel-Palade bodies (WPB) in the cytoplasm of vWf-positive cells than intimal ones (data not shown).

**Table 1:** An immunophenotypic analysis to correlate cell morphology and EC marker expression.

Constitutive EC markers	other cell types expressing it	requirement
vWf	platelets	
WPB	none	
CD31	platelets, lymphocytes, monocytes, neutrophils	
VE-cadherin	macrophages lining lymph nodes sinuses, trophoblasts	cell-cell junctions

The most relevant EC markers are vWf and VE-cadherin. vWf is also expressed by megakaryocytes and subsequently stored in platelets, but such cells are much smaller and devoid of nuclei. VE-cadherin is involved in EC adherent junctions and requires interaction between ECs to be stabilized at the plasma membrane. CD31 may be expressed by other cells. In both cases (intima and adventitial cells), the small polygonal cells are vWf-, CD31- and VE-cadherin-positive. The giant cells are most of the time vWf- and/or CD31- or VE-cadherin-positive. On the other hand, the elongated cells are rarely vWf-positive [22].

Being synthesized exclusively by ECs in the vessel wall (Table 1), vWf is a useful marker for the identification of these cells. This large glycoprotein is stored under a tubular multimerized form in WPB in resting cells, giving a granular pattern of reactivity. vWf, which is involved in the transport of Factor VIII, is released unfolded from the organelle and normally proteolysed by ADAMTS13 on secretion from ECs to mediate platelet adhesion to the sub-endothelium at sites of vascular injury [32]. A proportion of these long multimers may remain anchored to the activated endothelium, giving rise to vWf strings [33]. Fig 2B shows the various patterns of vWf staining in the isolated cell population. We observed reticular staining corresponding to vWf in the ER, immature WPB in the perinuclear region and granular staining corresponding to mature vWf scattered in the cytoplasm. Thus, the vWf staining appeared cytoplasmic and/or extracellular, and very heterogeneous in terms of intensity and subcellular localization. Nevertheless, vWf constitutes the only EC-specific marker amongst the nucleated cell types of the intima and adventitia. In addition, not all vWf-positive cells displayed WPB (Fig 2B). To validate vWf staining as a reliable EC marker despite its multiple patterns of presentation, confluent IECs and AECs, at P0 or P2 stages, were stained for other EC markers such as CD31 or VE-cadherin (Fig 2C). All cells positive for either CD31 or VE-cadherin did stain for vWf, either with or without WPB. Thus the VE-cadherin staining validated the vWf staining seen in the absence of WPB. However, CD31 is also a leukocyte marker and conclusive VE-cadherin staining is obtained only for confluent ECs. Taken together, these results indicated that small polygonal cells and most of the giant cells were ECs. We therefore selected the fractions where the combination of the two identified vWf-positive populations represented more than 90% of the total cell population after microscopic evaluation for further experiments.

We used vWf immunostaining to assess AEC isolation effectiveness. Because the adventitia is a thick and very heterogeneous tunica with VVs scattered throughout its entire thickness, a series of 16 consecutive extraction rounds was initially performed (8 to 10 min collagenase exposure), sampling sequentially the tissue from the external surface to the EEL (see Fig 1B). Each fraction could then be analyzed for EC content. Fixation was performed every other day and for two coverslips grown for the indicated period i.e., d1, d3, d5 or d7 (S2 Fig), starting arbitrarily from the most distal fractions and progressing towards the middle fractions. That way, EC content for each fraction and appropriate fixation time after cell isolation could be assessed. The criteria retained to evaluate the appropriate fixation time were: the number of cells (proliferation), cell spreading and vWf detection. From d1 until d3, the adherent cells remained round-shaped, being vWf positive but WBP negative but the high cell density (due to contaminating red blood cells (Fig 1E)) prevented their spreading, making them unsuitable for thorough immunofluorescence studies. From d5 onward (after daily removal of contaminating red blood cells by PBS washing), the cells had spread (and had started proliferating) and immunostaining analysis could be performed. ECs were identified by positive vWF staining and scored. All fractions contained ECs although in various proportions (S2 Fig, blue histogram), consistent with the variable density of VVs spanning the adventitial layer (see S1 Fig). WBP reformed, became detectable at d5 and 80% of ECs stained positive at d7 (S2 Fig, green histogram). After vessel injury or endothelial stress, vWf is released from ECs then re-synthesized by these cells. The data obtained thus likely reflect the stress induced by collagenase action and subsequent EC detachment, followed by vWf resynthesis and WPB reformation.

With this approach, the fraction with the highest EC enrichment could be selected (i.e., fraction 5). In addition, immunofluorescence analysis could be performed on P0 cells from d5 onward from the culture onset after plating cells on coverslip. Collectively, these data confirmed that vWf is a suitable and sufficient marker for ECs isolated through this procedure.

### Optimization of the isolation procedure: duration and rounds of enzymatic digestion and assessment of its efficiency

**Fig 3:**
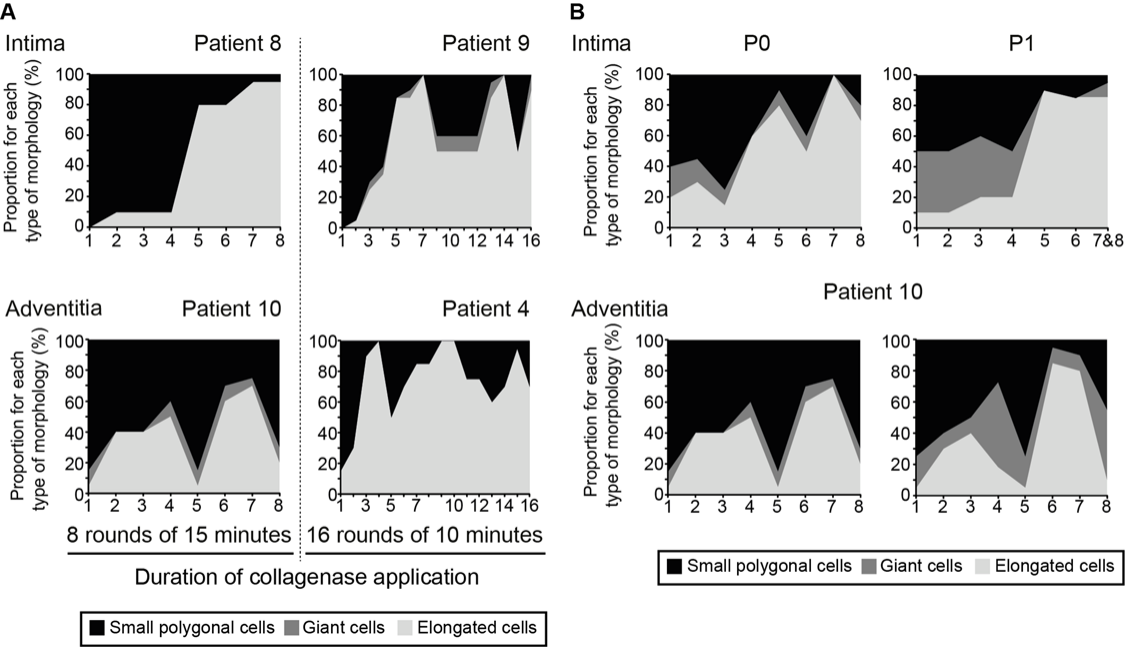
Optimization of the isolation procedure. (**A**) The graphs show the yield of EC-enrichment with the EC isolation procedure in optimized conditions. On the adventitial side, EC-enrichment was optimal when the collagenase was applied for 15 min and when 16 rounds of extraction were performed to obtain at least one EC-enriched fraction. On the intimal side, maximum EC-enrichment was obtained with 4 rounds of collagenase, each lasting 15 min. (**B**). Morphological analysis was also performed on P1 IECs and AECs, which could then be compared with that obtained at P0 cells to examine the effect of passaging and time in culture. The graphs show the proportion of vWf-positive cells. The general trend for the EC-contaminating cell ratio was similar for P0 and P1.

In the initial procedure to detach AECs (8 to 10 min collagenase exposure), the percentage of ECs in each fraction was variable, reaching up to 90% for some patients. We wondered if this percentage could be reproducibly improved, by reducing or increasing either the number of collagenase rounds or the duration of exposure to the collagenase. On the intimal side, we reasoned that an extended incubation time could release SMCs and leucocytes from the underlying sub-endothelium. On the adventitial side, the VVs are scattered throughout the tunica but concentrated in the so-called vasculogenic zone bordering on the EEL. It turned out that very few cells were extracted when the duration of proteolysis was less than 6 min. When a 10-min incubation period was applied, most fractions contained cells whereas all fractions contained cells when the tissue was exposed to collagenase for 15 min. Fig 3A compares the yield of EC-enrichment in optimized conditions for two distinct EC isolation procedures (*i*.*e*., 16 rounds 10 minutes versus 8 rounds 15 minutes) both applied on 2 different patients and highlight inter-patient heterogeneity. On the intimal side, maximum EC enrichment was obtained with 4 rounds of collagenase for 15 min and with 2 rounds of collagenase for 10 min. For AECs, it could not be predicted which of these fractions would show the best enrichment because this was dependent on the location of the VVs within a given adventitia. On this side, EC enrichment was optimal when the collagenase was applied for 15 min. As expected, the EC-enriched fraction(s) could be obtained at any of the 16 rounds of proteolysis, depending on the location of the VVs within the adventitia. More precisely, on average, 0.4 versus 1.1 fractions with 90–100% EC were obtained with 10 min versus 15 min of collagenase action for AEC when it was 1.6 versus 1.3 fractions for IEC. So, on the adventitial side, EC enrichment was optimal when the collagenase was applied for 15 min when for the intimal side there were not such a difference between 10 and 15 min on average.

To examine the behavior of the EC population with passaging and time in culture, morphological analysis was performed on P1 cells and was then compared with that of P0 cells. Fig 3B shows that the proportion of vWf positive cells, *i*.*e*., ECs, remained stable upon passaging. The putative contaminating adipocytes, SMCs, leucocytes and fibroblasts did not expand under the selective culture conditions applied throughout the procedure.

### Analysis of proliferating cells: selection of the EC-enriched fractions and validation of procedure

Most studies require a large number of cells. Once the protocol had been set up, the cell population was expanded after a second passage. This allowed us to perform biochemical studies (at P2 stage) to cross-validate the morphologic (performed at P0 and P1 stage by phase contrast) and phenotypic (performed at P0 and P2 stage by immunofluorescent staining) characterization of the isolated cells. Furthermore, western blot analysis allowed us to examine the changes in protein expression, such as endothelial versus mesenchymal markers, as a result of microenvironment-induced alterations of the endothelium during TAA progression. In this respect, chronic exposure to TGFbeta *in situ* was shown to induce alphaSMA and SM-MHC (early and intermediate mesenchymal markers, respectively) expression in these cells [34].

**Fig 4:**
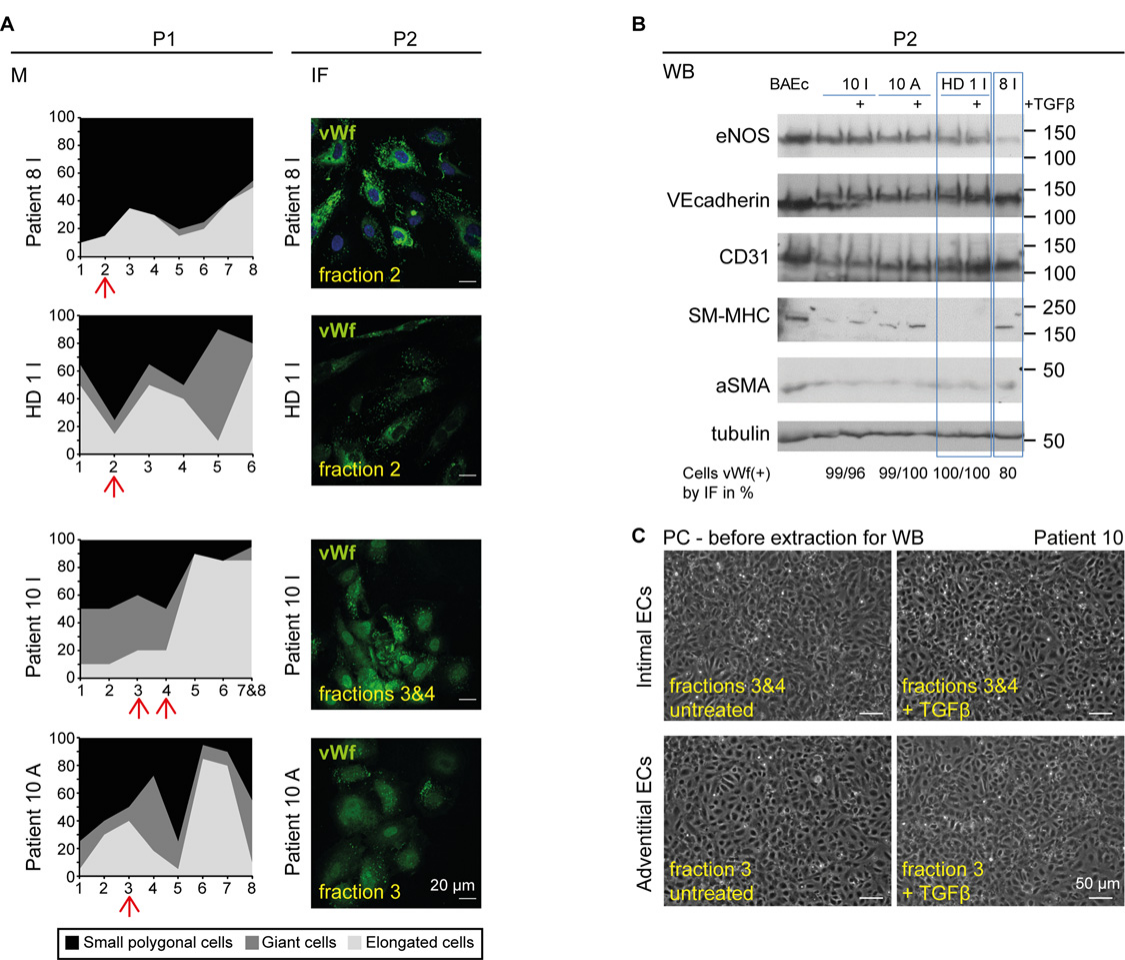
Analysis of proliferating cells: selection of EC-enriched fractions and validation of procedure. (**A**) The left-hand morphologic diagram (M) shows the fraction used (arrow) for western blot analysis in (B) (WB). The high percentage of ECs was confirmed in the corresponding vWf immunofluorescent staining (IF), performed at P2 (200 cells were scored). (**B**) P2 ECs isolated with the recommended procedure from three patients: 8I, 1I (HD) and 10I and A, were lysed and analyzed for three EC and two mesenchymal markers by SDS PAGE. (**C**) Representative phase contrast (PC) images of the cells from the fractions selected for patient 10, stimulated or not by TGFbeta, before cell lysis and western blot analysis, are shown. The biochemical studies performed at P2 cross-validate the morphologic (performed at P0 and P1 stage by phase contrast imaging) and immunophenotypic (performed at P0 and P2 stage by immunofluorescent staining) characterization of the isolated cells.

Representative fractions obtained from 3 patients (including one healthy donor, HD (1I)) are described in Fig 4A. Morphological assessment was 85% ECs for patient 8I fraction2, 85% ECs for patient 1I fraction 2, 80% for patient 10I fraction 3–4 and 75% ECs for patient 10A fraction3 (Fig 4B). We tested fractions estimated under 90% of ECs by this approach at P1 to determine whether it under- or over-estimated the percentage of ECs assessed by immunofluorescence analysis. At P2, the corresponding scores were 79, 100, 99 and 99% of vWf positive cells, respectively (Table 2). Clearly the morphological approach, in most cases, under estimated the EC-enrichment of the different fractions. Thus, by selecting, at P0, the fractions with 90% of small polygonal and giant cells (morphological criteria) for further analysis, we can be self-confident to find at least 90% of vWf positive cells at P2. By western blot (P2 cells), the three representative EC markers, namely eNOS, VE-cadherin and CD31 were detected, together with a weak signal for alphaSMA and SM-MHC (Fig 4B, WB panel).

**Table 2:** Immuno-phenotyping of the isolated cells processed for biochemical analysis.

Percentage of cells positive for an endothelial marker at P2 stage					
patient	Confluent cultures	Subconfluent cultures	Sparse cultures	medium	Endothelial marker used
**10 I wells 3 and 4**		99		serum	vWf
**10 I wells 3 and 4**		96		serum+TGFbeta	vWf
**10 A well 3**		99		serum	vWf
**10 A well 3**		100		serum+TGFbeta	vWf
**HD 1 I well 2**	97	99	100	w/o serum	vWf
**HD 1 Iwell 2**	99	100		serum	vWf
**HD 1 Iwell 2**		100		serum+TGFbeta	vWf
**8 I well 3**	75	79	79	serum	vWf
**8 I well 3**	78	82	75	serum	VE-cadherin

Data show the percentage of vWf-positive cells at P2 (determined by immunofluorescence), exposed or not to TGFbeta treatment, analyzed for EC and mesenchymal markers by western blot (Fig 4B). For each condition, 200 cells were analyzed.

The biochemical analysis was found matching the evaluation based on cell morphology and immuno-phenotypical analysis. In the example referred to as 1I (HD), (Fig 4A), 85% of the cells from fraction 2 displayed typical EC morphological characteristics at P1. At P2, 100% were positive for vWf by immunofluorescence (Table 2). The western blot analysis detected the three EC markers but not SM-MHC (Fig 4B). The alphaSMA signal was weak, as for all other samples, including the BAEc control at p5 (obtained from a commercial source and stated alphaSMA negative). The culture medium was expected to be free of TGFbeta. However, the ELISA assay detected a small amount of TGFbeta in the serum *i*.*e*., 5.37 ± 0.49 ng/ml total TGFbeta (n = 3) and 10.8 ± 4.35 pg/ml active TGFbeta (n = 4). When we examined the response of cells to the cytokine, it appeared that a 24h incubation period with 5ng/ml exogenous TGFbeta did not induce any more alphaSMA expression (Fig 4B). For the patient referred to as 10I and A, the cells were found to express the three EC markers (eNOS, VE-cadherin and CD31). This time, SM-MHC and, to a lesser extent alphaSMA, were detected (Fig 4B). We reasoned that this pattern could be a consequence of their exposure to TGFFbeta *in vivo*. Upon stimulation with exogenous TGFbeta for 24h *in vitro*, SM-MHC was induced but alphaSMA expression remained unaltered (Fig 4B). Likewise, the vWf expression pattern of cells isolated from 12 different patients was not impacted upon exogenous TGFbeta treatment (S3 Fig). Comparing the western blot patterns obtained with cells isolated from patient 8I (80% ECs) and 10I and A (99% ECs) confirmed mesenchymal contamination (clear SM-MHC/alphaSMA signal and weak eNOS signal) of the patient 8I fraction.

The combination of the three approaches described above indicated that the EC content was reliably evaluated using the sole morphological criteria at the P0 stage. Scoring the number of vWf positive cells revealed that the fraction of ECs in the total population at the P2 stage was actually higher than that predicted by this analysis (S3 Fig). Thus, the selection of the fraction where more than 90% of cells display the typical EC morphological characteristics at the P0 stage ensures the use of an EC-rich population after amplification. In addition, whereas alphaSMA expression was not informative, that of the mesenchymal marker SM-MHC could be attributed to EC phenotypic alterations, possibly as a result of *in vivo* exposure to TGFbeta.

### Functional assays

To validate our method, and because cells had been expanded *in vitro*, the functional properties of these ECs had to be addressed. ECs can be discriminated from other cells by their ability to form capillary-like structures when plated on basement membrane-like materials. ECs originating from any vascular bed are able to assemble spontaneously into tubules. Typically, Matrigel (a matrix-rich product prepared from Engelbreth–Holm–Swarm (EHS) tumor cells whose primary component is laminin) evokes EC tube formation within 24h. ECs first align themselves end-to-end, then elongate and a complex network of anastomosing cells can be observed after 16–24h.

**Fig 5:**
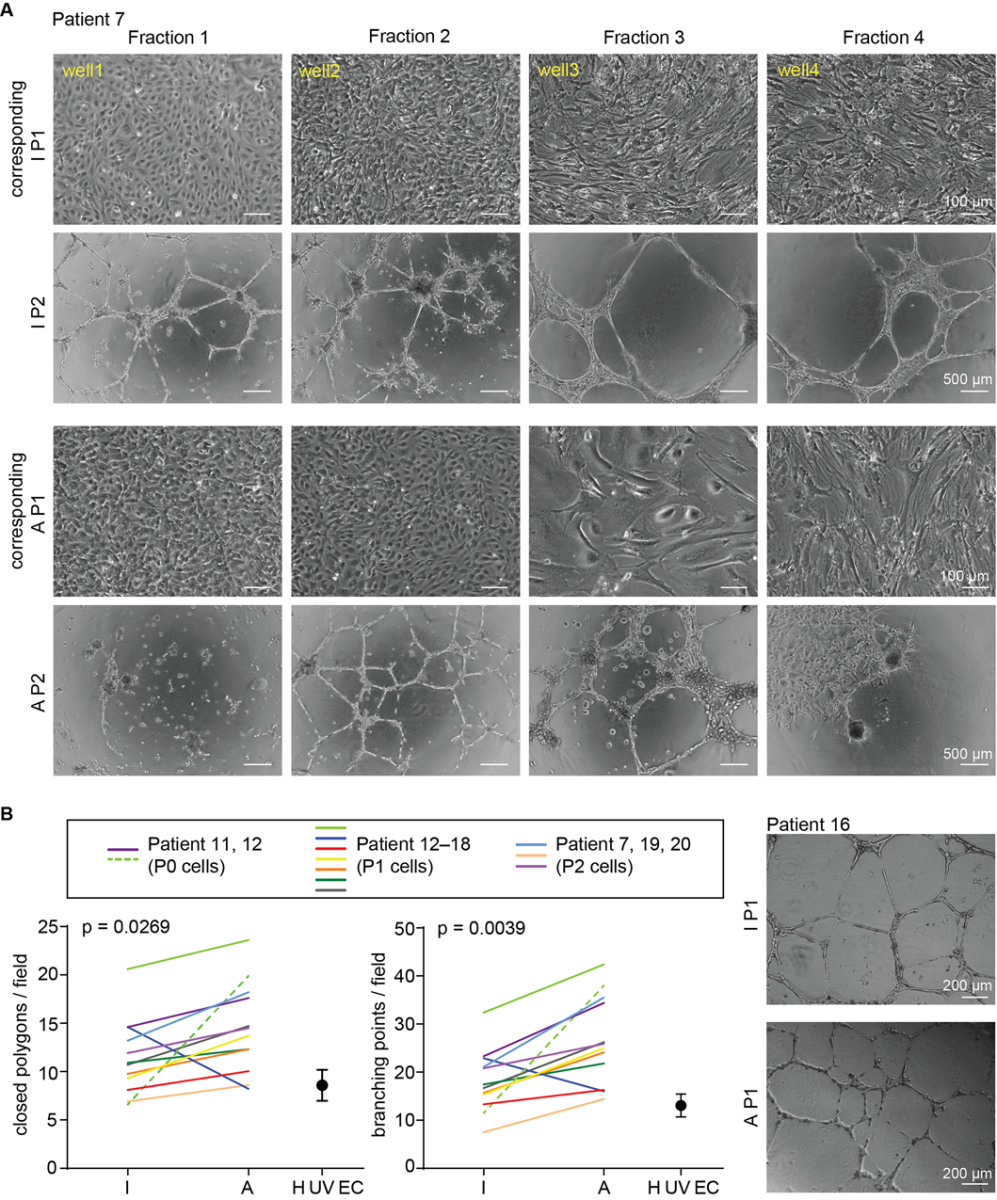
One representative experiment showing the ability of isolated cells to organize into tubular structures. (**A**) P1 cells were prepared from 4 consecutive fractions of cells isolated either on the intimal or the adventitial side of the same aorta (phase contrast images). Cells were amplified to test their performance in an *in vitro* angiogenesis assay. Resulting P2 cells were plated in 48-well plates coated with Matrigel. After 18h tubular structures had formed. Micrographs of the resulting network are shown (4x magnification). The EC-enriched fractions with the cobblestone appearance on plastic are those able to form tubes on Matrigel. In this example, the typical capillary-like structures were observed with IECs of fraction 1 and AECs from fraction 2. (**B**) Graphs showing the number of polygons or branching points, formed by IECs and AECs selected as the most EC-enriched fractions isolated from 11 different patients. AECsamples displayed more polygons (p = 0.0269) and more branching points (0.0039) than IEC ones. On the right panel, representative phase contrast micrographs showing the network formed by the selected (highest EC-enrichment) IEC and AEC fractions from patient 16, on Matrigel.

With this assay, tube formation was expected to occur with EC-rich fractions but not in the event of major contamination with other cells. The tube formation assay was performed with cells of the four intimal and adventitial fractions, all obtained from the same aorta. Cultured on tissue culture plastic, the cells from the first intimal fraction displayed a cobblestone appearance (Fig 5A). On Matrigel, the typical capillary-like structures were observed with cells of fraction1. From fractions 2 to 4, the proportion of mesenchymal-like cells increased. In these fractions, cells clearly became aligned and elongated but the number of them forming aggregates increased owing to contamination by non-EC cells. In cells extracted from the adventitial side, the second fraction was the one where they displayed the typical cobblestone appearance. In fractions 1 and 4, the proportion of elongated cells increased and in fraction 3, a mix of giant cells and mesenchymal-like cells was observed. The typical capillary-like structures were observed in cells from fraction 2. In fraction 3, some cells were clearly aligned and elongated but most of them were aggregated. In fraction 1, the cells were scattered while cell clusters and spread cells were observed in fraction 4.

As expected, the EC-enriched fractions with the cobblestone appearance when grown on plastic dishes, were those able to form tubes on Matrigel. In this example, the characterization of aneurysmal ECs would require to use the intimal fraction 1 for IECs and adventitial fraction 2 for AECs. When the angiogenic potential of the isolated cells was measured, the adventitial ECs were found to display significantly more polygons [35] than the intimal ones arising from the same aorta (Fig 5B). Similar but more significant results were obtained when the number of branching points [36] was scored (Fig 5B). These results confirm the isolation of two functionally distinct endothelial cell populations.

## Discussion

Aneurysm is associated with a complex remodeling of arteries that affects all their layers. The contribution of SMCs, fibroblasts, inflammatory cells and multipotent stem cells has been described [37,38] but the contribution of the ECs to TAA development remains largely unknown. Recent studies point to the endothelium as being important player in the pathogenesis of TAAs, although most of them were conducted with animal models. Although this represents an important and necessary starting point, the extent to which these data apply to human condition remains to be determined.

**Fig 6:**
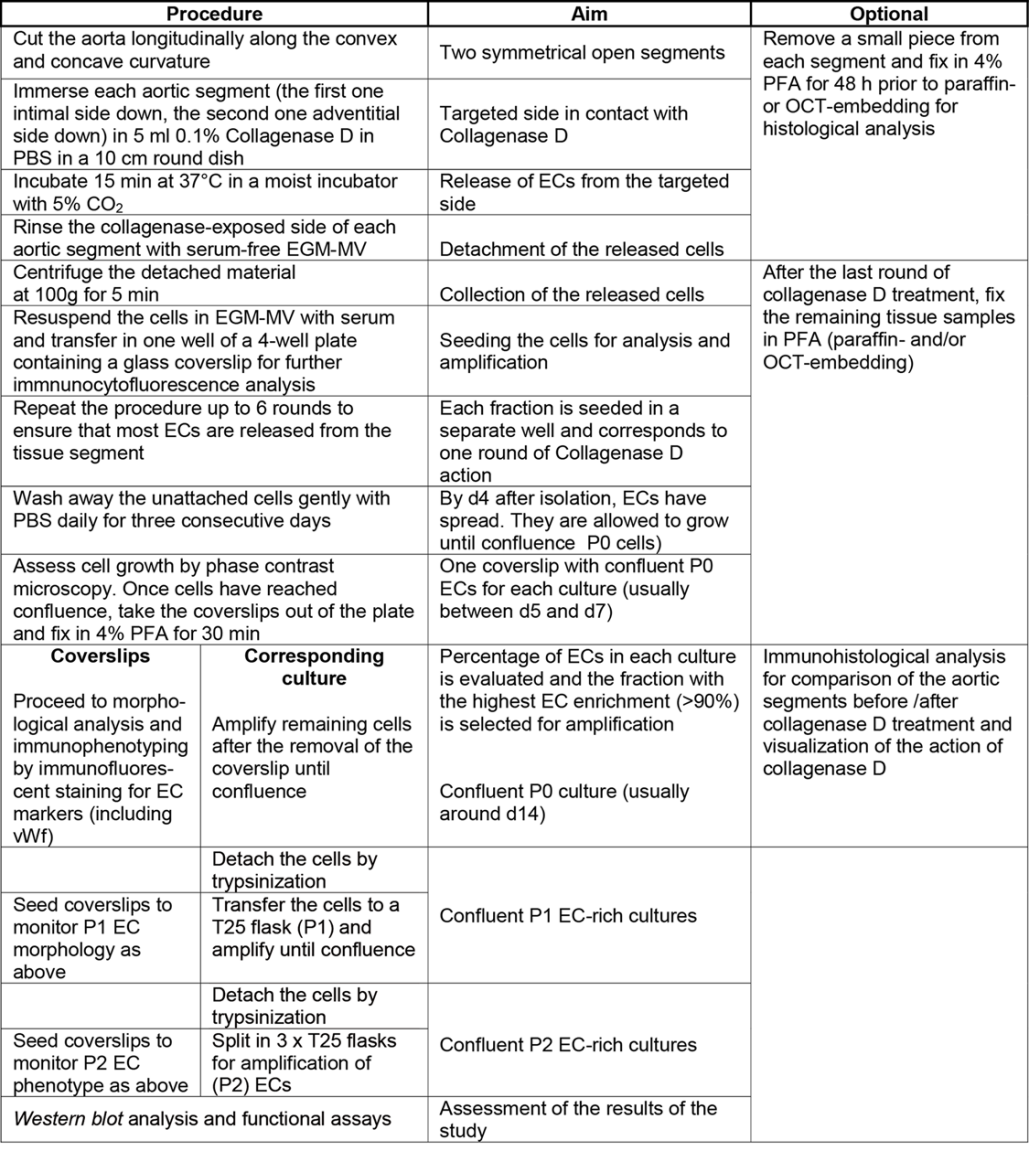
Procedure flow chart for the simultaneous isolation of ECs from the intima and adventitia of human aortic vessel segments.

The endothelium is not amenable to clinical examination and its thinness precludes conventional diagnostic imaging. To overcome these limitations, the isolation of ECs from diseased human vessels represents a valuable approach through which better understanding of TAA may be obtained. The results presented herein provide a reliable method for the isolation of IECs and AECs simultaneously (flow chart of the procedure summarized in Fig 6), which presents a number of improvements and advantages as discussed below.

A key improvement of this procedure is that it avoids the selection of a subpopulation of ECs. EC-enrichment was obtained from the isolated cell population in which ECs are the cells of the highest density, by plating the concentrated cell population on a limited surface, then by repetitively removing the non-attached contaminating cells. Another improvement of the technique is that it limits the phenotypic changes inherent to *in vitro* cultures. Immediately after the collagenase step, the freshly isolated cells were seeded together with the matrix debris resulting from collagenase action. By preserving part of the EC-associated ECM, the loss of the native environment is reduced and the stress minimized, so that EC may more promptly secrete the constituents of their basal lamina while other stromal components are provided in adequate culture medium [39]. In addition, with this method, the characteristics of the original EC phenotype (the P0 coverslip) and those of the tissue (embedded either in OCT or paraffin) were systematically integrated in the analysis. That way, the characteristics of cells at the P1 or P2 stages can be compared to those observed in cells of the P0 coverslip and/or those displayed by ECs in the native tissue. The procedure described herein allows selective isolation of VV ECs, which, to the best of our knowledge has never been reported. So far, investigations have been limited to the overall adventitial cell population isolated after elastase and collagenase digestion [40]. A number of studies now point at VV ECs as important player in a number of vascular diseases [14,41]. AECs become amenable to in vitro studies to characterize their specific features (see below). The phenotype and properties of IECs and AECs originating from the same vessel segment can be made. Finally, it should be pointed out that this method does not require expensive equipment or reagents [23] but common and usual tissue culture consumables and is therefore feasible in any tissue culture laboratory (Fig 6).

The endothelial origin of cells isolated with this procedure was established by the cobblestone appearance of a confluent cell monolayer, by the expression of vWf and/or the detection of WPB and by the ability of the cells to organize in tubules when plated on Matrigel. Most ECs were small, mononucleated and polygonal in shape but giant cells were occasionally found in fractions of either intimal or adventitial origin. Large multinucleated ECs are often found in primary cells of the human aorta [42] or in tissues [43] and are called multinucleated variant ECs. Their number increases with atherosclerosis grade and aging [44]. To our knowledge, this is the first description of the existence of giant microvascular cells. Being P0 cells, this phenotype more likely reflects aging but not degeneration induced by passaging. Rare elongated cells were occasionally found positive. These cells may correspond to those referred to as ‘sprouting cells’ by Schor and collaborators in BAEc primary cultures [31], who demonstrated that ECs are capable of undergoing a reversible phenotypic interconversion between two distinct morphologies. IECs and AECs were found morphologically indistinguishable.

Immunophenotypic studies revealed that vWf was the most reliable marker by providing a specific intracellular vesicular staining. IECs displayed the typical elongated WPB or, when lost owing to cell injury during the isolation procedure, were able to reform them upon culturing. The staining was less intense for AECs in agreement with the fact that the number of WPB decreases with vessel size and distance from the heart [45]. WPB were retained by IECs and AECs throughout the period of *in vitro* cultivation. From P0 to P2, there was no evidence of degenerative changes in cultured cells, which retained CD31, VE-cadherin and eNOS EC markers. The alphaSMA signal was detected in all samples, including the control commercial BAE cells in accordance with previous findings showing the existence of alphaSMA-positive ECs likely resulting from the stimulatory effect of TGFbeta contained in the serum. Although, alphaSMA-positive ECs have been detected *in situ* in the adult aortic endothelium [46], in our hands, alphaMA always stained negative using the immunohistochemistry approach. Likewise, the detection of the mesenchymal marker SM-MHC likely reflects the induction of an endothelial transformation (but also the presence of residual, contaminating mesenchymal cells). Indeed, alterations in the TGFbeta signaling pathway plays a decisive role in the pathogenesis of TAAs [47]. Total serum TGFbeta are found elevated in the entire spectrum of genetic aortic syndromes [48] and this may be reflected in circulating TGFbeta concentrations [49], as already shown for Marfan Syndrome [50]. ECs are able to undergo profound changes in phenotype, such as endothelial to mesenchymal transition (EndMT), defined by co-expression of SMC marker genes and the production of metalloproteases. The results presented herein indeed show variability in the pattern of SM-SMC expression in ECs isolated from TAAs, likely reflecting the induction of EndMT in the diseased *in vivo* environment. TGFbeta-induced transdifferentiation has been demonstrated for mature macrovascular cells [51] as well as microvascular cells [52]. The procedure proposed herein will make it possible to examine a plausible EndMT induced by TGFbeta on these two EC subsets *in vitro*.

Aortic dissection is often associated with the presence of an intimal tear, i.e., a rupture of the EC monolayer. IEC transformation may contribute to this process. However, a growing number of studies now support the opinion that VVs could also play a decisive role. Spontaneous rupture of VV leads to hematoma in the aortic wall, splitting of aortic wall layers and the formation of dissection without intimal tearing. VVs are found in the thoracic aorta from the 29^th^ lamellar unit outward, but not in the abdominal aorta [53]. This suggests that the thoracic aorta may have inherent properties that influence their susceptibility to TAA disease. In the course of these studies, we observed the frequent occurrence of medial VVs, detected by CD31 and vWf staining, in the vicinity of elastic lamina disruption and mucoid degeneration zones. These findings support the hypothesis of an increased likelihood that VVs will rupture in these weakened areas of the aortic media. Medial ingrowth of adventitial vessels may occur in response to elevated wall stress widening of the media. The occurrence of small intramural hemorrhages and focal interstitial edema around these new vessels could further aggravate the degenerative processes already underway in a faltering arterial wall. The method described herein is thus timely for exploring these hypotheses since, to the best of our knowledge, there have been no reports of VV ECs being isolated and cultured from the adventitial compartments of the aorta.

The results presented herein provide a reliable method for the isolation of IECs and AECs to characterize specific features of ECs that potentially contribute to aneurysmal diseases. Although the site-specific properties dependent on the signals from the diseased micro-environment may be lost (washed out in *in vitro* culture conditions), other vascular bed-specific properties should be retained [54], and those which are epigenetically fixed and mitotically stable will be conserved [55,56]. By analyzing cell-type specific gene expression rather than whole-organ tissue lysates, the method opens the way for transcriptomic (mRNA and miRNA), proteomic or metabolomic analysis of diseased IECs and AECs using expression profiling by microarray technologies. If needed, positive selection of the cells isolated by this method can be perform as an additional step to reach the level of purity required for this type of analysis. Comparison of protein, gene and miR expression profiles between aneurysmal and normal ECs may lead to the identification of novel candidate proteins associated with TAAs. The identification of other forms of EC dysfunction is expected to translate into measurable alterations that may allow the identification of reliable changes in circulating biomarkers. It will be possible to classify the types of aneurysms on the basis of the patterns of gene activity in the ECs. This will help the pharmaceutical community to develop more targeted and thus effective drugs as innovative therapeutic treatment strategies. In conclusion, this protocol provides a new approach for quantitative studies on the respective contribution of IECs and AECs in the pathogenesis and development of TAAs as well as other diseases where these cells are involved or suspected to be involved.

## Supporting Information

S1 Fig Structure of an aorta with TAA with representative images of the main ECM components. (**A**) The left-hand immunofluorescent image corresponds to a Sirius Red-stained section of aortic tissue from a patient with TAA analyzed under fluorescence microscopy. Collagen appears in red; elastic lamellae and fibers (autofluorescence) appear in green. Right, magnification of regions defined by white rectangle showing the inner part of the aortic media layer displaying a normal arrangement and distribution of elastic lamellae and collagen bundles and the outer part of the aortic media layer with presence of zones of disrupted elastic lamellae (arrow) with visible breakdown of collagen bundles (arrow head). (**B**) A micrograph showing ECs and mucoid basophilic material (proteoglycan pool) in a section of an aneurysmal aortic vessel segment after vWf immunostaining and Alcian blue and nuclear Red counter-staining under light microscopy. In the intima, the endothelium stains positive for vWf (in brown). In the outer part of the media, a range of SMCs is visible in the interspace between the parallel elastic lamellae, their nucleus appear in pink and mucoid, basophilic material (Alcian blue stained GAG) appears in blue. A medial vasa vasorum (VV) immunostained with vWf localizes in the close vicinity of an area of mucoid degeneration (*) devoid of SMCs. Adventitial VVs positive for vWf are visible along the EEL. (**C**) Schematic diagram of the structural organization of the intima including the distribution of various types of cells within aortic intima based on the description of the adult aorta wall by Stary and colleagues [1]. Besides ECs, the major components of the intima, a few other cell types have been observed by transmission electronic microscopy. Their proportion is depending on the intimal thickness. The IEE is the boundary between the intima and the media. (**D**) Micrograph showing the vascular distribution of the tunica adventitia revealed by hematoxylin and eosin (H and E) staining. The image illustrates the vast heterogeneity of this layer. In the higher magnification views in the bottom panel, arrows point to VVs, adipocytes, peripheral adrenergic nerve endings. Red blood cells are visible in the luminal space. The outer part of the media is visible.
(TIF)

S2 Fig Validation of the selection based on the morphological criteria by vWf immunostaining and optimization of the isolation procedure. (**A**) Immediately after collection, each of the 16 crude fractions was seeded on one coverslip. Cells were fixed, two at a time and every other day from day1 to day 7. At d1 or d1 and d2, cells had not spread and immunofluorescence did not provide any valuable information. At d3 or d4, cells had spread and vWf staining allowed to score the percentage of positive cells and to assess the presence of WPB. Bright staining likely reflects vWf re-synthesis following EC damage. WPB were reformed at d7. With this approach, the fraction with the highest EC enrichment could be selected.
(TIF)

S3 Fig Validation of EC-enriched fraction selection procedure by vWf staining, regardless the presence or absence of TGFbeta. The graph shows the percentage of cells positive for vWf at P2, with or without TGFbeta treatment, determined by immunofluorescence for 12 patients (IECs and AECs from 5 patients and AECs from two additional patients). p>0.5.
(TIF)
