# Supplementary figures and images for: A Methodology for Concomitant Isolation of Intimal and Adventitial Endothelial Cells from the Human Thoracic Aorta
Source: PLoS One. 2015 Nov 24;10(11):e0143144. doi: 10.1371/journal.pone.0143144 (PMC4658207; doi:10.1371/journal.pone.0143144)

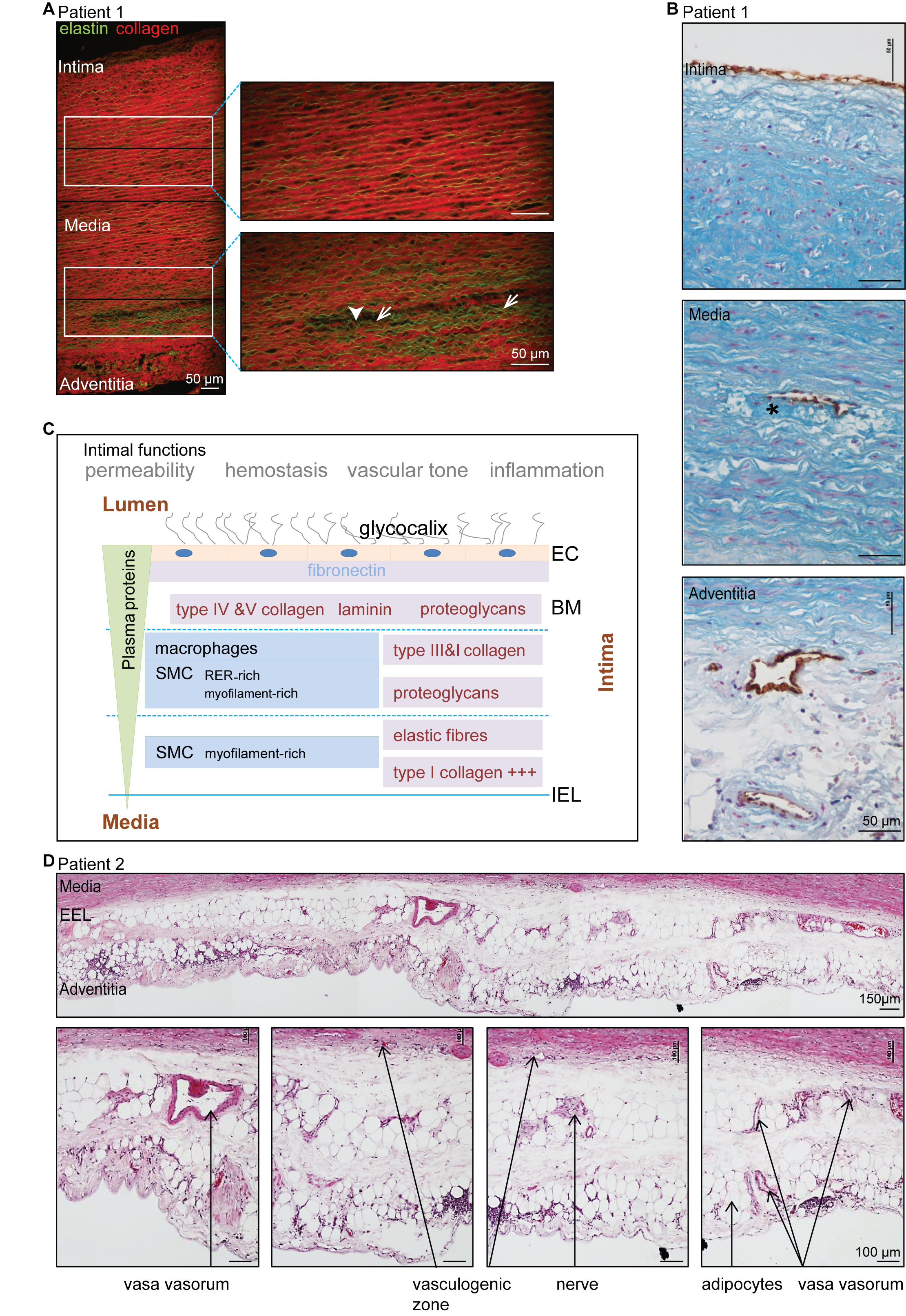

Supplement: S1 Fig — (A) The left-hand immunofluorescent image corresponds to a Sirius Red-stained section of aortic tissue from a patient with TAA analyzed under fluorescence microscopy. Collagen appears in red; elastic lamellae and fibers (autofluorescence) appear in green. Right, magnification of regions defined by white rectangle showing the inner part of the aortic media layer displaying a normal arrangement and distribution of elastic lamellae and collagen bundles and the outer part of the aortic media layer with presence of zones of disrupted elastic lamellae (arrow) with visible breakdown of collagen bundles (arrow head). (B) A micrograph showing ECs and mucoid basophilic material (proteoglycan pool) in a section of an aneurysmal aortic vessel segment after vWf immunostaining and Alcian blue and nuclear Red counter-staining under light microscopy. In the intima, the endothelium stains positive for vWf (in brown). In the outer part of the media, a range of SMCs is visible in the interspace between the parallel elastic lamellae, their nucleus appear in pink and mucoid, basophilic material (Alcian blue stained GAG) appears in blue. A medial vasa vasorum (VV) immunostained with vWf localizes in the close vicinity of an area of mucoid degeneration (*) devoid of SMCs. Adventitial VVs positive for vWf are visible along the EEL. (C) Schematic diagram of the structural organization of the intima including the distribution of various types of cells within aortic intima based on the description of the adult aorta wall by Stary and colleagues [1]. Besides ECs, the major components of the intima, a few other cell types have been observed by transmission electronic microscopy. Their proportion is depending on the intimal thickness. The IEE is the boundary between the intima and the media. (D) Micrograph showing the vascular distribution of the tunica adventitia revealed by hematoxylin and eosin (H and E) staining. The image illustrates the vast heterogeneity of this layer. In the higher magni [file pone.0143144.s001.tif]

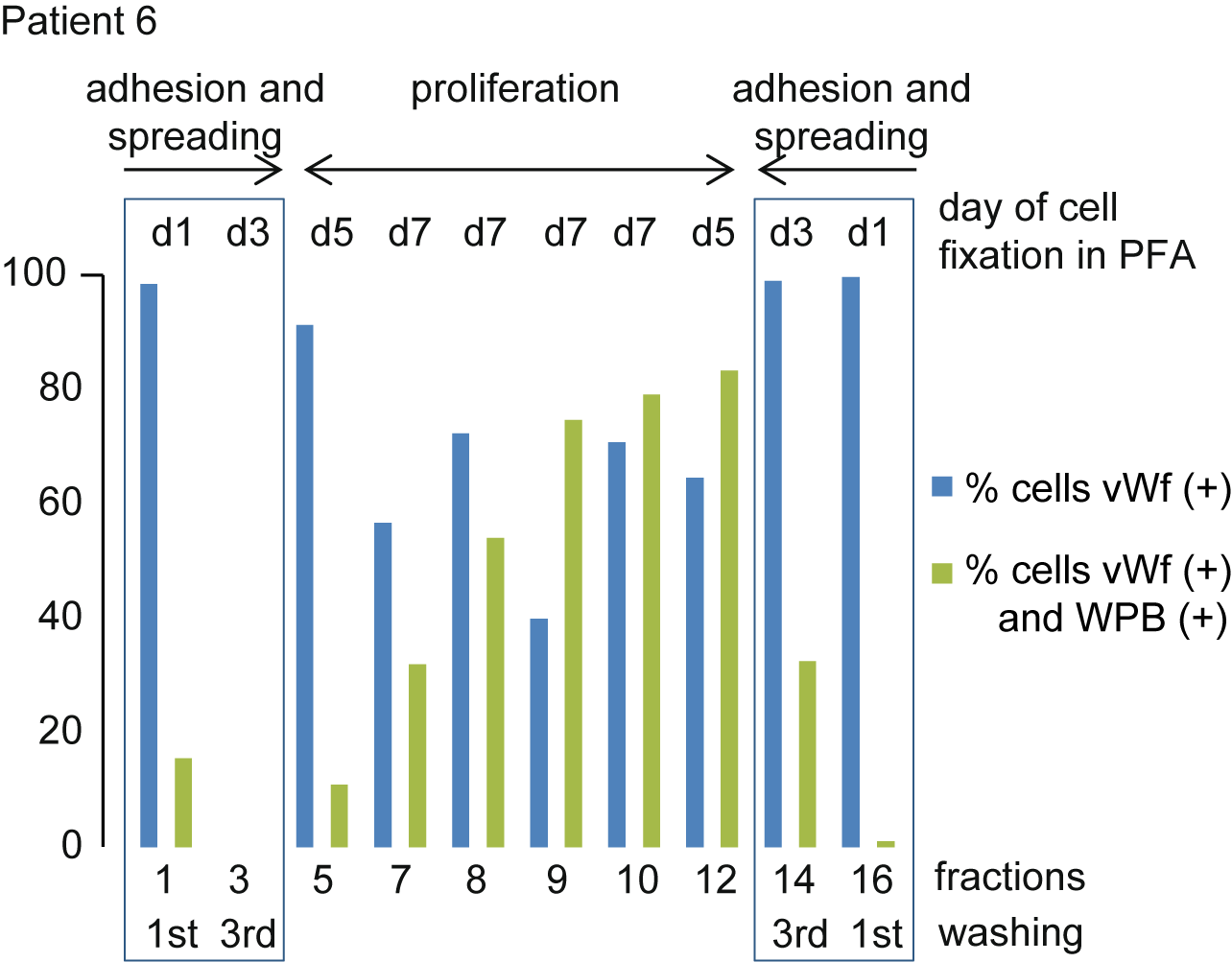

Supplement: S2 Fig — (A) Immediately after collection, each of the 16 crude fractions was seeded on one coverslip. Cells were fixed, two at a time and every other day from day1 to day 7. At d1 or d1 and d2, cells had not spread and immunofluorescence did not provide any valuable information. At d3 or d4, cells had spread and vWf staining allowed to score the percentage of positive cells and to assess the presence of WPB. Bright staining likely reflects vWf re-synthesis following EC damage. WPB were reformed at d7. With this approach, the fraction with the highest EC enrichment could be selected. (TIF) [file pone.0143144.s002.tif]

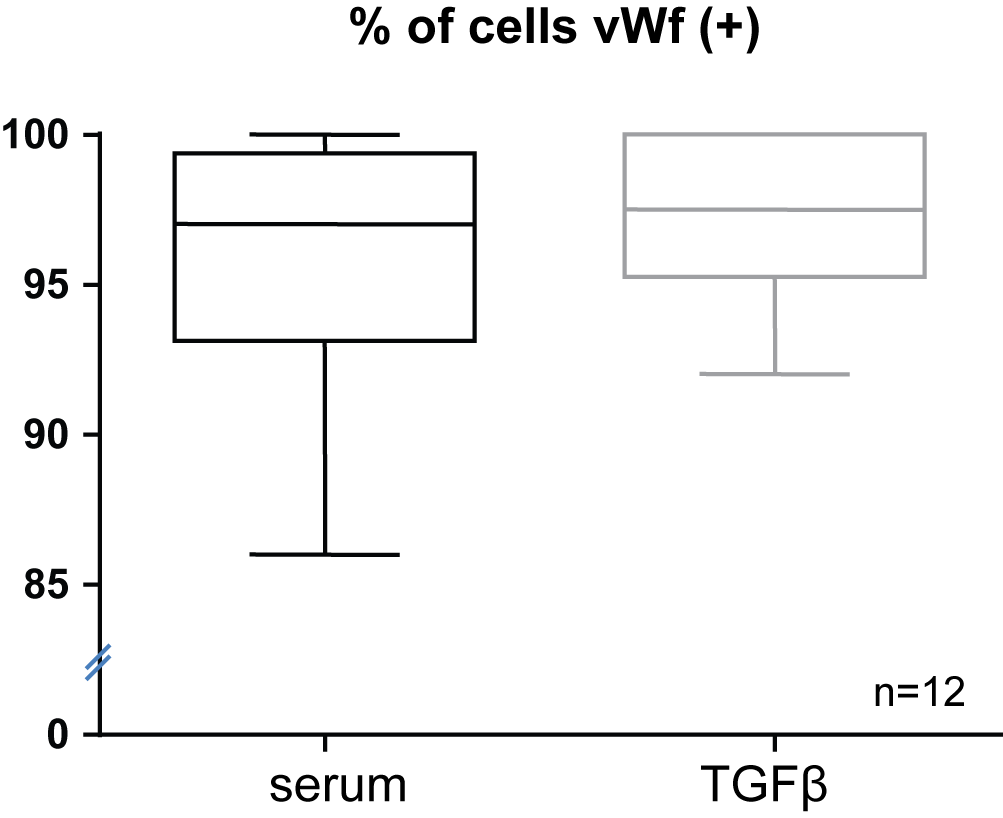

Supplement: S3 Fig — The graph shows the percentage of cells positive for vWf at P2, with or without TGFbeta treatment, determined by immunofluorescence for 12 patients (IECs and AECs from 5 patients and AECs from two additional patients). p>0.5. (TIF) [file pone.0143144.s003.tif]
